# Supplementary material for: MetaRibo-Seq measures translation in microbiomes
Source: Nat Commun. 2020 Jun 29;11:3268. doi: 10.1038/s41467-020-17081-z (PMC7324362; doi:10.1038/s41467-020-17081-z)
Supplement: Supplementary file 10 — Supplementary Data 7 [file 41467_2020_17081_MOESM10_ESM.zip › File2/Confidence_High_Taxonomy/98262_out.krona.html]

Javascript must be enabled to view this page.

members
magnitude
magnitudeUnassigned
count
unassigned
taxon
rank

98262\_out

4

superkingdom
4
2

1239
phylum
4

186801
4
class

186802
order
4

4
family
216572

459786
genus
4

876091

SRS142890\_contig\_number\_42042SRS143598\_contig\_number\_23462SRS148424\_contig\_number\_32668
3
species

1
species

SRS098827\_contig\_number\_5027
1897011
